# Oblique Lateral Interbody Fusion vs. Minimally Invasive Transforaminal Lumbar Interbody Fusion for Lumbar Spinal Stenosis: A Retrospective Cohort Study

**DOI:** 10.3389/fmed.2022.829426

**Published:** 2022-05-19

**Authors:** Quan-You Gao, Fei-Long Wei, Tian Li, Kai-Long Zhu, Ming-Rui Du, Wei Heng, Fan Yang, Hao-Ran Gao, Ji-Xian Qian, Cheng-Pei Zhou

**Affiliations:** ^1^Department of Orthopedics, Tangdu Hospital, Fourth Military Medical University, Xi'an, China; ^2^School of Basic Medicine, Fourth Military Medical University, Xi'an, China

**Keywords:** lumbar spinal stenosis, oblique lateral interbody fusion (OLIF), Minimally Invasive Transforaminal Lumbar Interbody Fusion (MIS-TLIF), postoperative improvements, retrospective study

## Abstract

**Background:**

Minimally invasive transforaminal lumbar interbody fusion (MIS-TLIF) is an effective surgical option for lumbar spinal stenosis (LSS) with spondylolisthesis. However, few studies have discussed oblique lateral interbody fusion (OLIF) with MIS-TLIF.

**Objective:**

To evaluate postoperative improvements, complications, and reoperation rates between patients with LSS undergoing OLIF or MIS-TLIF.

**Methods:**

We retrospectively studied 113 LLS patients who underwent OLIF (53) or MIS-TLIF (60) with percutaneous pedicle screw fixation between January 2016 and December 2018. We measured estimated blood loss, operative time, hospital stay, reoperation, and complication incidence, visual analog scale (VAS), Oswestry Disability Index (ODI), Japanese Orthopedic Association (JOA), and Short Form-36 (SF-36) scores, discal and foraminal height and lumbar lordotic angle.

**Results:**

The mean age was 58.81 ± 0.9 years. The TLIF group had increased operation time, blood loss, and hospital stays (*p* = 0.007, 0.001, and 0.016, respectively). Postoperatively, VAS and ODI scores significantly decreased while JOA and SF-36 scores significantly increased. The postoperative differences in main outcomes between the groups were insignificant (all *p* > 0.05). The OLIF group had the lowest rates of overall (9.8% OLIF vs. 12.9% MIS-TLIF), intraoperative (3.9% OLIF vs. 4.8% MIS-TLIF), and postoperative complications (5.9% OLIF vs. 8.1% MIS-TLIF), but the differences were insignificant (*p* = 0.607, 0.813, and 0.653, respectively). The reoperation rate did not differ significantly (3.8% OLIF vs. 3.3% MIS-TLIF) (*p* = 0.842). OLIF restored disc height (74.4 vs. 32.0%), foraminal height (27.4 vs. 18.2%), and lumbar lordotic angle (3.5 vs. 1.1%) with greater success than did MIS-TLIF.

**Conclusion:**

Patients undergoing OLIF with LSS improved similarly to MIS-TLIF patients. OLIF restored disc height, foraminal height and lumbar lordotic angle more successfully than did MIS-TLIF.

## Introduction

Lumbar spinal stenosis (LSS) is a degenerative condition that can be caused by bony, discal, or ligamentous structures (1). A potential catalyst of low back pain and disability (2, 3), LSS is the most common reason for spinal surgery in patients over 65 years of age (2). When conservative treatment does not relieve symptoms, surgical intervention is warranted (1, 4).

Lumbar interbody fusion (LIF) is an effective treatment for various spinal diseases, including protrusion of the lumbar intervertebral disc, LSS and lumbar spondylolisthesis (5). There are many approaches to performing LIF, each with their own advantages and disadvantages. Minimally invasive transforaminal lumbar interbody fusion (MIS-TLIF) and oblique lumbar interbody fusion (OLIF) is frequently used for LIF (6). MIS-TLIF seeks to minimize soft tissue and muscle damage, whereas the traditional open TLIF is characterized by extensive surgical exposure (7, 8). OLIF is increasingly used to treat degenerative lumbar disease due to the reduced tissue damage and blood loss associated with this method (9). OLIF expands the inner volume of the spinal canal using indirect decompression (10, 11). However, comparative studies on these two technologies are limited (6). Hence, the efficacy of indirect decompression as an alternative to direct decompression for LSS has not been determined yet.

The purpose of this study was to compare the outcomes between MIS-TLIF and OLIF surgery in LSS patients. We also evaluated the differences in complications and reoperation rates between the two approaches.

## Materials and Methods

### Patient Selection

This retrospective cohort study was conducted at Tangdu Hospital, Fourth Military Medical University. It was performed in accordance with the Declaration of Helsinki and approved by the hospital's ethics committee. The work was reported in line with the STROCSS criteria (12). We included 113 patients who were diagnosed with LSS and underwent either MIS-TLIF or OLIF surgery between January 2016 and December 2018. Lumbar spine dynamic radiographs were routinely performed in all patients to assess lumbar spine stability. Use CT and MRI to evaluate the stenosis of the lumbar spinal canal, the distance between the inferior vena cava and veins/iliac vessels and the psoas muscle to determine the surgical plan and decide whether OLIF can be performed. The inclusion criteria were as follows: LSS due to neurogenic claudication; Central stenosis or lateral stenosis who need surgery; low-grade (Meyerding grade I–II, L2~S1) isthmic spondylolisthesis or degenerative spondylolisthesis; and imaging findings consistent with the symptoms of LSS. The exclusion criteria were trauma, active infection, malignant tumors, spinal deformity, previous lumbar fusion, multi-segment fusion, high-grade (Meyerding grade III or IV) isthmic spondylolisthesis or degenerative spondylolisthesis.

### Surgical Procedure

#### OLIF

**Figure 1:**
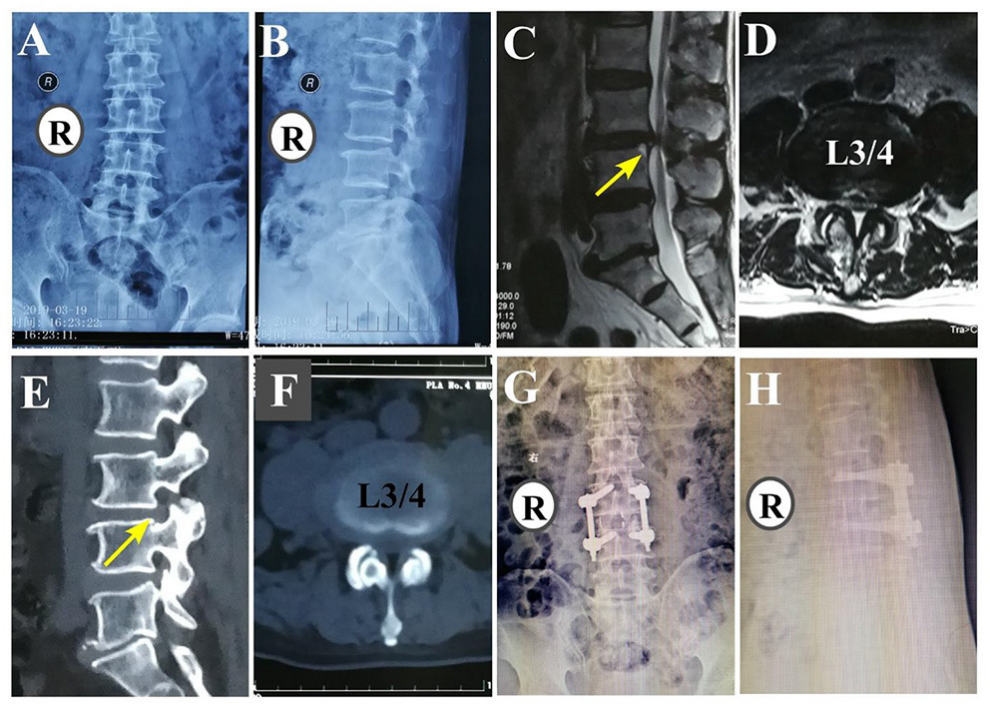
A 79-year-old man with low back pain and weakness of both lower extremities with OLIF (L3/4). **(A,B)** Preoperative anteroposterior and lateral fluoroscopy. **(C,D)** Preoperative sagittal and axial MRI. **(E,F)** Preoperative sagittal and axial CT **(G,H)** Postoperative anteroposterior and lateral fluoroscopy.

Overall, 53 patients underwent OLIF surgery (6, 13). The patient was placed in the right lying position after induction of general anesthesia. The external oblique, internal oblique, and transverse abdominal muscles were cut in the direction of the fibers. In the retroperitoneal space, we performed blunt dissection through the plane between the retroperitoneal fat and lumbar muscle in order to access the lumbar spine while protecting both the lumbar muscle and plexus. Once the target disc was exposed, we performed subtotal discectomy to prepare the vertebral endplate. A wide cage was chosen to prevent sinking. Subsequently, a cage filled with allogeneic bone was placed, and its position was confirmed by fluoroscopy. Four percutaneous pedicle screws were inserted into the vertebral body, and bilateral rods were used for fixation. A rubber drainage tube was placed, and the incision was closed in layers. Representative LSS patients who underwent OLIF are shown in Figure 1.

#### MIS-TLIF

**Figure 2:**
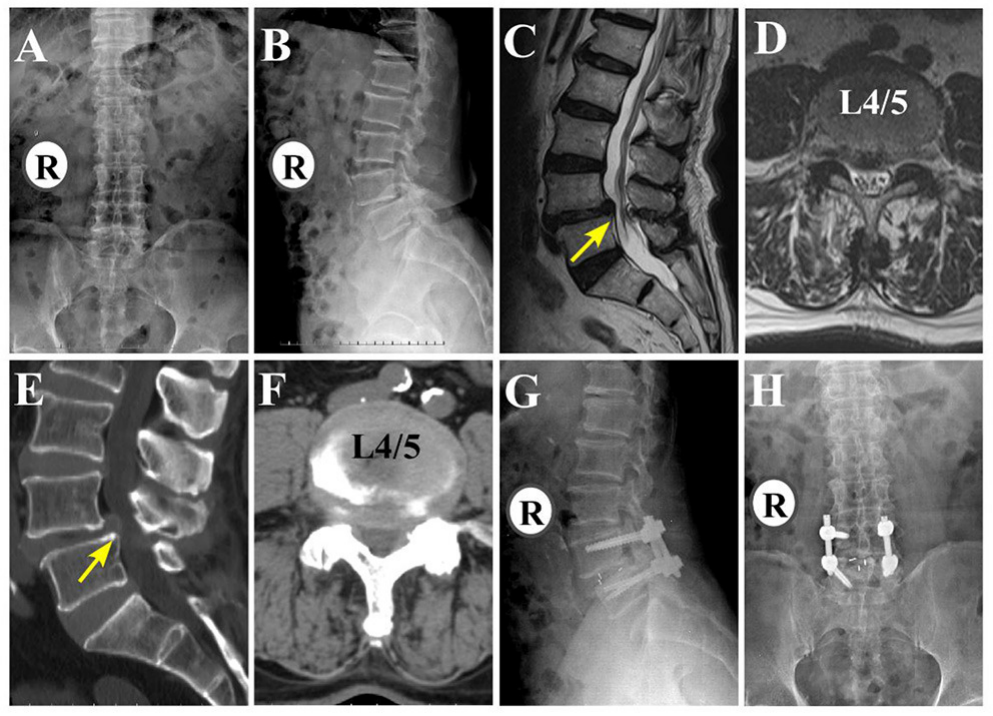
An 80-year-old man with low back pain and left lower extremity radiculopathy with MIS-TLIF (L4/5). **(A,B)** Preoperative anteroposterior and lateral fluoroscopy. **(C,D)** Preoperative sagittal and axial MRI **(E,F)** Pre-operative sagittal and axial CT. **(G,H)** Postoperative anteroposterior and lateral fluoroscopy.

Overall, 60 patients underwent MIS-TLIF surgery (7, 14). A lateral incision was made 2–3 cm from the midline. The length of this incision was 2–2.5 cm, and it was longitudinally located between the multifidus and longissimus muscles. Intervertebral body preparation, bone transplantation, and cage placement were performed using either a series of dilators and tubular retractors or pedicle screw retractors. For fusion, bilateral discectomy was performed using a unilateral channel approach. A suitable cage was placed, and its position was confirmed by fluoroscopy. Pedicle screw fixation was performed either before or after TLIF. The screws were placed percutaneously using guide wires and dilators. A rubber drainage tube was inserted, and the incision was closed in layers. Representative LSS patients who underwent MIS-TLIF are shown in Figure 2.

#### Postoperative Management

After surgery, anti-infective preventive measures were prescribed. Furthermore, patients were asked to perform active ankle dorsiflexion and passive straight leg raises. When the fluid volume was <50 mL/24 h, the drainage tube was pulled out within 48 h after surgery. For 2 to 3 days after surgery, patients were asked to wear orthoses for out-of-bed activities.

#### Outcome Measures

We assessed the following patient characteristics: estimated blood loss, operative time, hospital stay, reoperation rate, and complications, such as dural tear (15), nerve injury and vascular injury, deep tissue infection, and instrumentation failure. Before surgery and at the last follow-up, the following information was recorded: visual analog scale (VAS), Oswestry Disability Index (ODI), Japanese Orthopedic Association (JOA), and Short Form-36 (SF-36) scores (16); discal and foraminal height and lumbar lordotic angle (17).

#### Statistical Analysis

Statistical analyses were performed using the SPSS statistical software package (version 23.0; IBM, Chicago, IL). An independent *t*-test was used to compare continuous data between the OLIF and MIS-TLIF groups. The paired *t*-test was used to compare the continuous data obtained preoperatively and postoperatively. Categorical variables were assessed using the Chi-square test. For all analyses, statistical significance was defined as *p* < 0.05.

## Results

A total of 113 patients (51 males and 62 females) were included in this study. The mean follow-up period was 25.816 ± .06 months and 26.655 ± .79 months in the OLIF and MIS-TLIF groups, respectively (*p* = 0.454). The mean age was 58.81 ± 0.9 years. The most commonly affected spinal segment was L4/5 in both groups. The clinical characteristics of the included patients are shown in Table 1. At baseline, there was no significant difference between the preoperative clinical characteristics of the patients in the OLIF and MIS-TLIF groups (Table 1). The MIS-TLIF group experienced increased operation time, blood loss, and hospital stays (*p* = 0.007, 0.001, and 0.016, respectively; Table 1).

**Table 1:** Clinical characteristics of included patients.

**Variables**	**OLIF(*N* = 53)**	**Mis-TLIF (*N* = 60)**	**Difference (95%CI)**	***P*-value**
**Age**	58.42 ± 9.98	59.23 ± 11.66	NA	0.691
**Gender**			NA	0.850
**Male**	23 (43.4%)	28 (46.7%)		
**Female**	30 (56.6%)	32 (53.3%)		
**BMI**	23.74 ± 2.77	24.67 ± 3.16	NA	0.102
**Operative segments**			NA	0.924
**1**	38 (67.9%)	45 (75%)		
**2**	12 (22.6)	12 (20%)		
**3**	3 (9.4)	3 (5%)		
**Disc height (mm)**	8.15 ± 0.93	8.50 ± 0.96	NA	0.53
**Foraminal height (mm)**	17.23 ± 0.87	18.69 ± 1.24	NA	0.001^*^
**Lumbar lordotic angle (** **°** **)**	37.72 ± 1.19	38.06 ± 1.12	NA	0.12
**Operative time(min)**	144.26 ± 30.94	171.67 ± 56.28	−27.40 (−44.65 to −10.15)	0.002^*^
**Blood loss (mL)**	176.23 ± 68.45	361.83 ± 125.19	−185.61 (−223.93 to −147.28)	0.001^*^
**Hospital stay (day)**	12.26 ± 3.49	15.07 ± 7.38	−2.80 (−5.02 to −0.60)	0.013^*^

* *Statistically significant (P < 0.05); Plus–minus values are means ±SD; NA denotes not applicable*.

**Figure 3:**
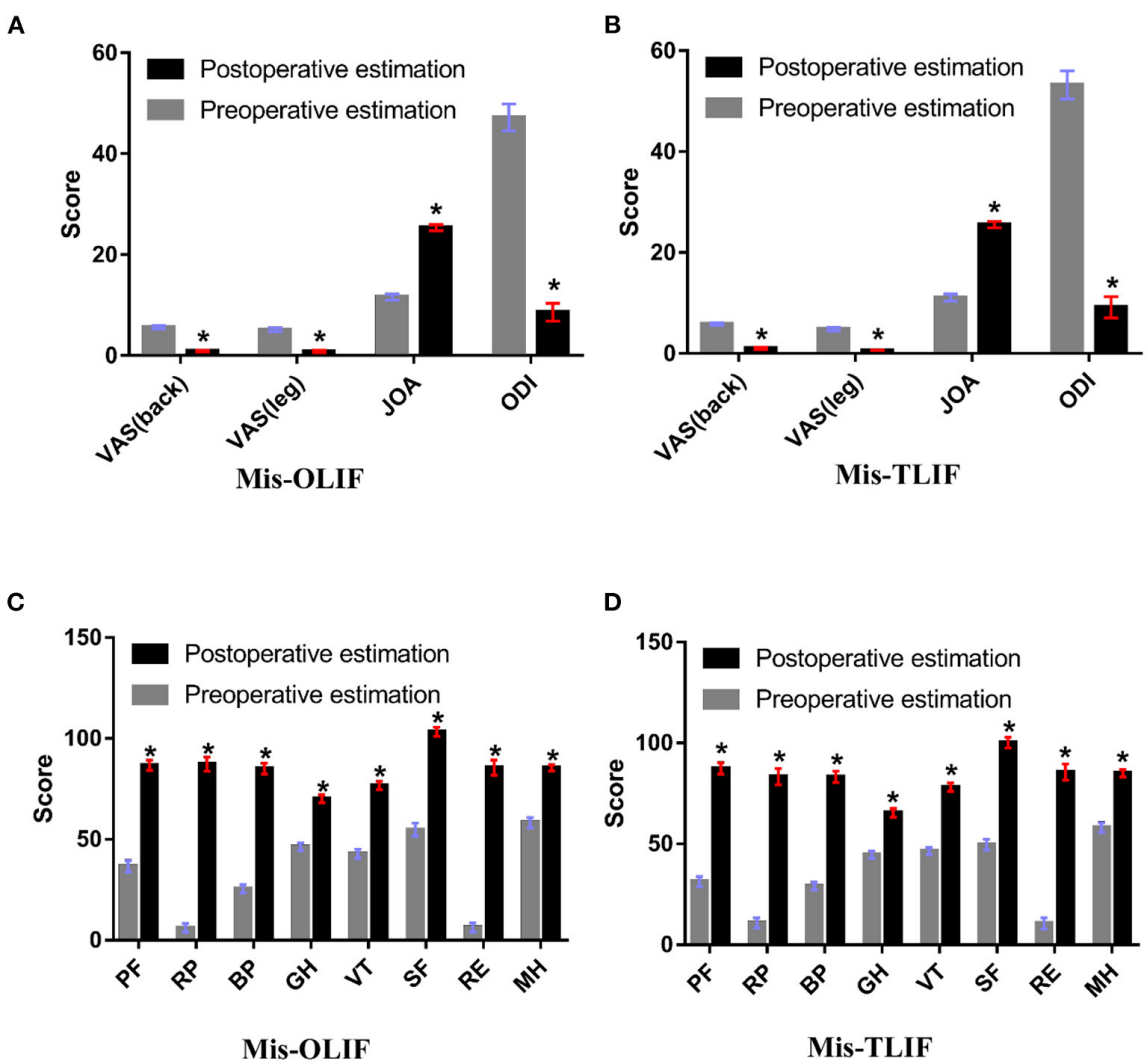
**(A,B)** Pre- and postoperative VAS (leg), VAS (lumbar), JOA, and ODI scores between the groups; **(C,D**) Pre- and postoperative SF-36 scores in between the groups. PF, Physical functioning; RP, role limitations-physical; BP, bodily pain; GH, general health; VT, vitality; SF, social function; RE, role limitations-emotional; and MH, mental health.

As shown in Table 2, there were no significant differences in main outcomes, including VAS, JOA, ODI, and SF-36 between the OLIF and MIS-TLIF groups at baseline. VAS and ODI scores decreased postoperatively, while JOA and SF-36 scores increased significantly (Figure 3). There were no significant postoperative differences in the main outcomes between the two groups (all *p* > 0.05, Table 3).

**Table 2:** Preoperative estimation of mean values of main outcomes.

**Outcome**	**OLIF**	**Mis-TLIF**	***P*-value**
**VAS (lumbar)**	5.64 ± 2.30	5.82 ± 1.84	0.654
**VAS (leg)**	5.13 ± 2.74	4.83 ± 2.66	0.558
**JOA**	11.62 ± 4.42	11.10 ± 5.63	0.587
**ODI**	47.22 ± 19.22	53.22 ± 21.61	0.124
**SF-36**			
**PF**	36.60 ± 20.42	31.33 ± 19.63	0.165
**RP**	6.13 ± 16.19	10.83 ± 19.18	0.165
**BP**	25.49 ± 15.21	29.08 ± 14.43	0.201
**GH**	46.34 ± 13.97	44.68 ± 14.90	0.545
**VT**	42.83 ± 16.42	46.42 ± 13.44	0.205
**SF**	54.72 ± 23.28	49.58 ± 20.45	0.215
**RE**	6.29 ± 16.09	10.56 ± 21.69	0.243
**MH**	58.26 ± 18.41	58.07 ± 18.29	0.955

^*^Statistically significant (P < 0.05); Plus–minus values are means ±SD. PF, Physical functioning; RP, role limitations-physical; BP, bodily pain; GH, general health; VT, vitality; SF, social function; RE, role limitations-emotional; and MH, mental health.

**Table 3:** Postoperative estimation of mean values of main outcomes.

**Outcome**	**OLIF**	**Mis-TLIF**	**Difference (95%CI)**	***P*-value**
**VAS (lumbar)**	0.911 ± 0.08		−0.11 (−0.59 to 0.37)	0.650
**VAS (leg)**	0.851 ± 0.54	0.551 ± 0.03	0.30 (−0.18 to 0.78)	0.223
**JOA**	25.364 ± 0.38	25.534 ± 0.83	−0.17 (−1.90 to 1.55)	0.841
**ODI**	8.621 ± 2.79	9.171 ± 6.39	−0.55 (−6.08 to 4.98)	0.844
**SF-36**				
**PF**	86.691 ± 8.50	87.422 ± 1.91	−0.72 (−8.33 to 6.90)	0.852
**RP**	87.262 ± 4.81	83.333 ± 0.76	3.93 (−6.57 to 14.44)	0.460
**BP**	85.041 ± 9.54	83.222 ± 1.86	1.82 (−5.95 to 9.59)	0.643
**GH**	70.161 ± 4.20	65.371 ± 6.72	4.80 (−1.02 to 10.63)	0.105
**VT**	76.691 ± 4.73	78.081 ± 6.08	−1.39 (−7.16 to 4.39)	0.636
**SF**	103.301 ± 6.10	100.212 ± 0.39	3.09 (−3.82 to 10.01)	0.377
**RE**	85.532 ± 7.35	85.563 ± 0.91	−0.02 (−10.96 to 10.92)	0.852
**MH**	85.431 ± 0.52	85.001 ± 3.67	0.43 (−4.16 to 5.03)	0.955

^*^Statistically significant (P < 0.05); Plus–minus values are means ± SD. PF, Physical functioning; RP, role limitations-physical; BP, bodily pain; GH, general health; VT, vitality; SF, social function; RE, role limitations-emotional; and MH, mental health.

As shown in Table 2, the OLIF group had lower overall (9.8% OLIF vs. 12.9%) and intraoperative complication rates (3.9% OLIF vs. 4.8%) compared to those in MIS-TLIF group, but the differences were not statistically significant (*p* = 0.607 and 0.813, respectively, Table 4). Among the patients in the OLIF group, one patient experienced thigh numbness and another experienced hematoncus. There were three patients with intraoperative complications (two with dural tears and one with nerve injury) in the MIS-TLIF group. There were no statistical differences in the postoperative complication (5.9% OLIF vs. 8.1% MIS-TLIF) and reoperation rates (3.8% OLIF vs. 3.3% MIS-TLIF) (*p* = 0.653 and 0.842, respectively, Table 1).

**Table 4:** Complication and reoperation of included patients.

**Outcome**	**OLIF**	**Mis-TLIF**	***P*-value**
**All complications**	5 (9.8%)	8 (12.9%)	0.607
**Intraoperative complication**	2 (3.9%)	3 (4.8%)	0.813
**Postoperative complication**	3 (5.9%)	5 (8.1%)	0.653
**Reoperation**	2 (3.8%)	2 (3.3%)	0.842

^*^Statistically significant (P < 0.05).

**Figure 4:**
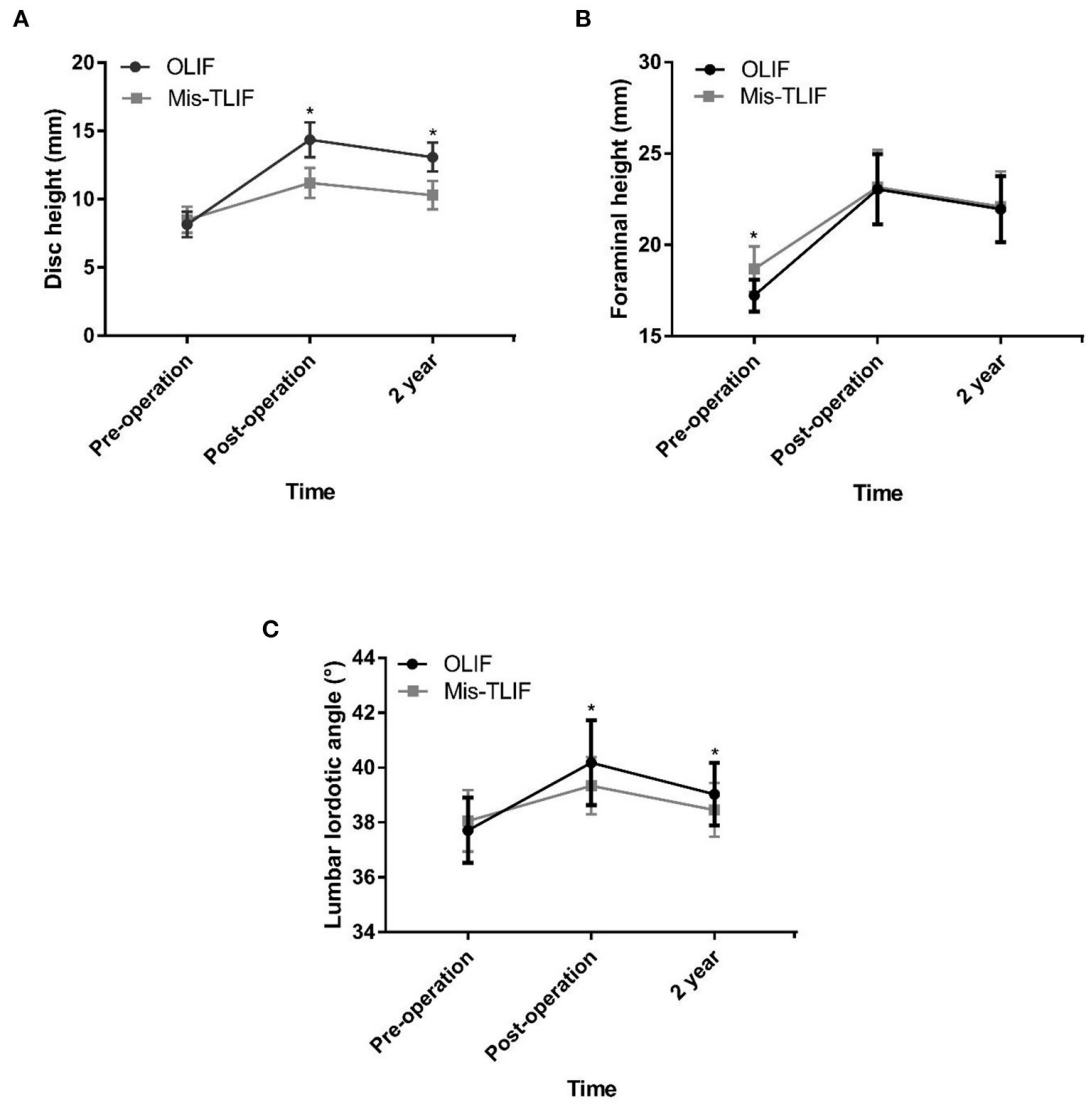
**(A–C)** Disc height (mm), foraminal height (mm), and lumbar lordotic angle (°) improvements between the OLIF and MIS-TLIF groups.

Disc height (mm) was significantly restored from a preoperative mean of 8.15 to a postoperative one of 14.21 (*P* < 0.001) in the OLIF group, with an average postoperative increase of 74.4%. In the MIS-TLIF group, the disc height was significantly restored from a mean of 8.49 at baseline to 11.19 immediately following surgery (*P* < 0.001), with an average postoperative increase of 32.0%. The improvement in disc height in the OLIF group was significantly greater than that in the MIS-TLIF group, and the difference was statistically significant (Figure 4A). Foraminal height (mm) was higher in the MIS-TLIF group than in the OLIF group (*p* = 0.001, Table 1); however, there was no statistical difference in foraminal height between the two groups following surgery, thereby indicating that OLIF was superior to MIS-TLIF in terms of restoring foraminal height (27.4 vs. 18.2%, Figure 4B). Additionally, OLIF was better than MIS–TLIF at restoring the physiological lumbar lordotic angle (3.5 vs. 1.1%, Figure 4C).

## Discussion

This retrospective trial included 113 patients with LSS, of which some also had degenerative spondylolisthesis. The purpose of this study was to compare the prognosis for LSS patients when using MIS-TLIF or OLIF, both of which are minimally invasive spinal surgeries. The results revealed that the clinical efficacies of MIS-TLIF and OLIF were not statistically different. This finding was consistent with those of a previous study (18). Compared with the more conventional MIS-TLIF approach, OLIF was associated with superior increases in disc height, foraminal height, and physiological lumbar lordotic angle at the final follow-up.

OLIF is used to achieve indirect decompression (19), whereas MIS-TLIF achieves decompression directly (6). The volume of the spinal canal can be enlarged through OLIF by reducing the area and thickness of the ligamentum flavum, thereby relieving nerve compression. Our results revealed that OLIF could significantly reduce patient pain while also improving function (Figures 2A,C). Khechen et al. retrospectively compared postoperative outcomes between primary MIS-TLIF and MIS-TLIF with revision decompression. Their results showed that both methods provided satisfactory clinical efficacy (20). Our results are consistent with these findings (Figures 2B,D). We found that the blood loss in the OLIF group was lower than that in the MIS-TLIF group, which may be a result of indirect decompression removing the need to enter the spinal canal. This explanation has been proposed in previous studies (18–21). Patients in the OLIF group had shorter hospital stays than those in the MIS-TLIF group because OLIF does not affect the function of the lumbar spine joints, allowing a faster return to physical activity (22).

OLIF was conducted through the window between the abdominal major vessels and the anterior border of the psoas muscle (23) using a method reported in 2012 (11). Silvestre et al. found that only seven patients (3.9%) had complications related to lumbar plexus injury or psoas muscle weakness, and all of their patients made a complete recovery (11). Sato et al. reported the clinical results of 20 patients with degenerative spondylolisthesis who were treated using OLIF. Thigh pain or numbness was present in two patients (10%), although this disappeared within 2 weeks after the surgery (24). Only 1 patient had thigh numbness, but this was relieved within 5 days after the surgery. This was also observed in another study (23). One of the main problems of OLIF is the large abdominal blood vessel injury that occurs during surgery. However, Silvestre et al. reported that vascular injury of the iliac or iliolumbar vein occurred in only three patients (1.7%) and that this could be repaired successfully even with a small incision (11). There were no patients with complications related to injury of the major vessel in our study, a finding which is reflected in a previous study (23). In our study, two patients (3.8%) experienced intraoperative complications (one had thigh numbness and another experienced hematoncus requiring subsequent surgery). When using OLIF, we achieved satisfactory results in patients with LSS caused by a decrease in the height of the intervertebral space by ≥1/2 compared with the adjacent segment, ligamentum flavum hypertrophy, and herniated intervertebral disc; however, when LSS was caused by a decrease in the height of the intervertebral space by <1/3, or when articular process hyperplasia was present, relatively poor results were achieved using OLIF. This indicates that indirect decompression is not effective in decompression caused by small joint hyperplasia. OLIF holds more advantages in terms of restoring spinal balance, which might affect the overall spine balance and reduce the risk of certain diseases, such as long-term degeneration of the adjacent segments (25). OLIF can only perform indirect decompression, so it is a contraindication for patients with high-grade central canal stenosis. In addition, patients with spondylolisthesis more severe than Meyerding grade II are not candidates for OLIF because the two vertebral end plates do not have sufficient “overlap” to support the intervertebral joint on the oblique trajectory of the intervertebral disc space (26). Contraindications to MIS-TLIF include extensive epidural scarring, arachnoiditis, active infection and associated nerve roots (which may prevent access to the disc space) and patients with osteoporosis (26).

MIS-TLIF is a muscle-sparing surgical approach for lumbar spine fusion where the surgeon enters via the natural cleavage plane between the multifidus and longissimus muscles using a method referred to as the paraspinal approach (27, 28). Wong et al. found that deep wound infection (0 vs. 5.6%) and reoperations (8.3 vs. 20.4%) were significantly lower in the MIS-TLIF group (144 cases) than in the open TLIF group (*n* = 54 cases) (29). A recent meta-analysis by Phan et al. further demonstrated that MIS-TLIF cases had a lower infection rate compared with that in open TLIF (1.2 vs. 4.6%, *p* < 0.001) (30). However, there were no cases of deep wound infection in their study. A meta-analysis Tan et al. found that the rate of dural tears in MIS-TLIF group was 3.0% (95% CI: 1.5–5%) (31). Two patients had dural tears which were covered with a gelatin sponge. Two patients underwent surgery again (one due to internal fixation failure and another due to cage subsidence). The patients recovered well after the second surgery. With respect to complications, the overall complication incidence of MIS-TLIF (12.9%) was slightly higher than that of OLIF (9.8%). However, the results were not statistically significant. Previous study reported the complication rate was significantly higher in the OLIF group than in the MI-TLIF group (32). However, the recent meta-analysis reported that there was no statistically significance in complication between OLIF group and MI-TLIF group (33). Dural tear and nerve root injury are the most common approach-related complications of MIS-TLIF due to the narrow transforaminal passage (33). In contrast, thigh numbness often occurs after OLIF, which may be caused by damage to the psoas nerve branch and continuous traction (33).

This study was, however, not without its limitations. We did not assess the continuous changes in patient outcomes beyond those between the baseline and final follow-up. Our sample size was not large enough to draw a definitive conclusion. The learning curves of the surgeon with regard to OLIF and MIS-TLIF may be a potential limitation, especially about estimated blood loss, surgical complications, and improvement rate. This phenomenon has been referred to as result bias (9, 34–38). Additionally, our patients did not show any serious complications, which reflects the high level of experience of the surgeons to whom this steep learning curve no longer applies.

The current study alleviates this bias by enrolling patients with similar characteristics, BMIs, histories of previous lumbar spine surgery and diagnoses in both groups, but randomized prospective trials should be conducted in the future in order to compare the MIS-TLIF and OLIF methods.

In conclusion, the clinical outcomes of OLIF appear to be similar to MIS-TLIF. OLIF was better than MIS-TLIF at restoring disc height, foraminal height, and physiological lumbar lordotic angle. In summary, using OLIF when appropriately indicated can result in the same surgical effects as MIS-TLIF.

## Data Availability Statement

The original contributions presented in the study are included in the article/supplementary material, further inquiries can be directed to the corresponding author/s.

## Ethics Statement

The studies involving human participants were reviewed and approved by Ethics Committee of Tangdu Hospital, Fourth Military Medical University. The patients/participants provided their written informed consent to participate in this study. Written informed consent was obtained from the individual(s) for the publication of any potentially identifiable images or data included in this article.

## Author Contributions

Q-YG, F-LW, TL, H-RG, J-XQ, and C-PZ contributed substantially to the conception and design of the work, acquisition and interpretation of data, and the drafted work. All authors contributed to the article and approved the submitted version.

## Funding

This work was supported by grants from the National Natural Science Foundation of China (No. 81871818) and Tangdu Hospital Seed Talent Program (F-LW) and Social Talent Fund of Tangdu Hospital (No. 2021SHRC034). The funding body had no role in the design of the study, data collection, analysis, and interpretation and in writing the manuscript.

## Conflict of Interest

The authors declare that the research was conducted in the absence of any commercial or financial relationships that could be construed as a potential conflict of interest.

## Publisher's Note

All claims expressed in this article are solely those of the authors and do not necessarily represent those of their affiliated organizations, or those of the publisher, the editors and the reviewers. Any product that may be evaluated in this article, or claim that may be made by its manufacturer, is not guaranteed or endorsed by the publisher.
